# Ajwa Date (*Phoenix dactylifera L*.) Extract Inhibits Human Breast Adenocarcinoma (MCF7) Cells *In Vitro* by Inducing Apoptosis and Cell Cycle Arrest

**DOI:** 10.1371/journal.pone.0158963

**Published:** 2016-07-21

**Authors:** Fazal Khan, Farid Ahmed, Peter Natesan Pushparaj, Adel Abuzenadah, Taha Kumosani, Elie Barbour, Mohammed AlQahtani, Kalamegam Gauthaman

**Affiliations:** 1 Biochemistry Department, Faculty of Science, King Abdulaziz University, Jeddah, Saudi Arabia; 2 Center of Excellence in Genomic Medicine Research, King Abdulaziz University, Jeddah, Saudi Arabia; 3 Center of Innovation in Personalized Medicine, King Fahd Medical Research Center, King Abdulaziz University, Jeddah, Saudi Arabia; 4 Biochemistry Unit, King Fahd Medical Research Center, King Abdulaziz University, Jeddah, Saudi Arabia; 5 Department of Agriculture, Faculty of Agricultural and Food Sciences, American University of Beirut (AUB), Beirut, Lebanon; Winship Cancer Institute of Emory University, UNITED STATES

## Abstract

**Introduction:**

*Phoenix dactylifera L* (Date palm) is a native plant of the Kingdom of Saudi Arabia (KSA) and other Middle Eastern countries. Ajwa date has been described in the traditional and alternative medicine to provide several health benefits including anticholesteremic, antioxidant, hepatoprotective and anticancer effects, but most remains to be scientifically validated. Herein, we evaluated the anticancer effects of the Methanolic Extract of Ajwa Date (MEAD) on human breast adenocarcinoma (MCF7) cells *in vitro*.

**Methods:**

MCF7 cells were treated with various concentrations (5, 10, 15, 20 and 25 mg/ml) of MEAD for 24, 48 and 72 h and changes in cell morphology, cell cycle, apoptosis related protein and gene expression were studied.

**Results:**

Phase contrast microscopy showed various morphological changes such as cell shrinkage, vacuolation, blebbing and fragmentation. MTT (2-(4,5-dimethylthiazol-2-yl)-2,5-diphenyltetrazolium bromide) assay demonstrated statistically significant dose-dependent inhibitions of MCF7 cell proliferation from 35% to 95%. Annexin V-FITC and TUNEL assays showed positive staining for apoptosis of MCF7 cells treated with MEAD (15 mg and 25 mg for 48 h). Flow cytometric analyses of MCF7 cells with MEAD (15 mg/ml and 20 mg/ml) for 24 h demonstrated cell cycle arrest at 'S' phase; increased p53, Bax protein expression; caspase 3activation and decreased the mitochondrial membrane potential (MMP). Quantitative real time PCR (qRT-PCR) analysis showed up-regulation of *p53*, *Bax*, *Fas*, and *FasL* and down-regulation of *Bcl-2*.

**Conclusions:**

MEAD inhibited MCF7 cells *in vitro* by the inducing cell cycle arrest and apoptosis. Our results indicate the anticancer effects of Ajwa dates, which therefore may be used as an adjunct therapy with conventional chemotherapeutics to achieve a synergistic effect against breast cancer.

## Introduction

Breast cancer is the most common and leading cause of cancer related mortality among women globally [1]. In 2012, more than 464,000 new cases were diagnosed with breast cancer in the European Union (EU) and in the United States of America (USA) [2]. It was estimated that there will be 249,260 new cases of female breast carcinoma in the year 2016 [3, 4]. Breast cancer is the most common cancer of women in the Kingdom of Saudi Arabia (KSA), and there is a steady increase in breast cancer patients from 1152 in 2008 to 1473 in 2010 [5]. Due to high mortality and the associated side effects of chemotherapy and/or radiotherapy, cancer patients often seek alternative forms of therapies such as natural or herbal medicines [6]. This has led to an increased interest and active search for novel anticancer agents from natural products. Historically, many potent anticancer agents such as vincristine, vinblastine, paclitaxel, etoposide, camptothecin, topotecan, and doxorubicin were derived from plants [7]. Vinblastine, vincristine, vinorelbine, vindesine, taxol, etoposide, topotecan and irinotecan are well used in clinical practice as combinational therapy for many malignancies [8, 9].

*Phoenix dactylifera L*. is an important fruit of desert regions of the Middle East, North Africa, India, Pakistan, Southern Europe and South America [10]. Ajwa dates are soft, delightful berries that have basal white lines on black exocarp and are chiefly cultivated in Al-Madina Al-Munawwarah, KSA [11]. Ajwa dates are generally the most preferred as they are rich in carbohydrates, proteins, minerals, dietary fibers, vitamins, fats and have high nutritional value compared to other varieties of dates [10]. Ajwa date also contains numerous phytochemicals such as flavonoids, glycosides, phytosterols and polyphenols [12]. These phytochemicals are claimed to have hepatoprotective [13], nephroprotective [14], antioxidant and anti-inflammatory properties [12]. Polyphenol and digested extract of Ajwa dates are reported to inhibit Caco-2 colon cancer cell line *in vitro* [15]. Inhibition of sarcoma-180 in mice by carbohydrate (1→3)-β-D-glucan isolated from Lebanon dates further highlights the anticancer property of dates [16]. The anticancer effects of date fruits on human breast cancers, hitherto remain unexplored. As such, in the present study, we attempt to evaluate the anti-cancer effects of the Methanolic Extract of Ajwa Dates (MEAD) on human breast adenocarcinoma (MCF7) cell *in vitro*.

## Materials and Methods

### Preparation of Ajwa date extract

Ajwa dates that were fresh, ripe, medium sized, fleshy, soft, and have basal white lines on the black exocarp was used in the present study. Ajwa dates, originally cultivated in the city of Al-Madina Al-Munawwarah were purchased from “Tamar Market” of Al-Madina Al-Munawwarah, KSA. MEAD was prepared using previously described protocol with slight modification [17]. Briefly, the edible part of date fruit was manually separated, freeze dried and coarsely powdered using pestle and mortar. The powdered contents were then extracted in methanol (1:3 ratio, weight to volume) on a shaking incubator (Human Lab, Gyeonggi-Do, Korea) at 25°C for 48 h. The resultant extract was filtered using Whatman No. 1 filter paper and centrifuged at 4000 g for 15 min and vaporized under low pressure at 45°C using Rotavapor^®^R-300 (Buchi, Flawil Switzerland). The supernatant was separated and freeze dried using a lyophilizer (ilShin Biobase Europe, The Netherlands). The freeze dried powder was aliquoted and stored at -80°C until use in the experiments.

### Cell culture

The human breast adenocarcinoma cell line (MCF7) was purchased from American Type Culture Collection (ATCC, Manassas, VA, USA). Institutional ethical approval was obtained for use of cancer cells in this study. The MCF7 cells were maintained Dulbecco’s minimal essential medium (DMEM), low glucose (Invitrogen, Carlsbad, CA) supplemented with 10% fetal bovine serum and 0.01 mg/ml bovine insulin and 1% penicillin-streptomycin, (Invitrogen, Carlsbad, CA) at 37°C in a humidified atmosphere of 95% air and 5% CO_2_.

### Cell imaging

MCF7 cells were cultured in 24-well tissue culture plates (Beckton, Dickinson, Franklin Lanes, NJ) at a seeding density of 2×10^4^ cells/well. After overnight attachment, the cells were treated with different concentrations (5, 10, 15, 20 and 25 mg/ml) of MEAD and the untreated cells served as control (S1 Methods). The cells were cultured for up to 72 h under standard culture conditions of 37°C in a humidified atmosphere of 95% air and 5% CO_2_. Changes in cell morphology were imaged using inverted phase contrast microscope (Nikon Instruments, Tokyo Japan).

### Cell proliferation assay

The effects of MEAD on MCF7 proliferation/inhibition was analyzed using MTT reagent [18]. Briefly, 2×10^4^ cells/well were seeded in 24-well plates and allowed to attach overnight. The cells were then treated with different concentrations (5, 10, 15, 20 and 25 mg/ml) of MEAD and incubated at 37°C in a humidified atmosphere with 5% CO_2_ for up to 72 h. Following the treatment period, MTT reagent (5 mg/ml) in 100 μl of fresh culture medium was added to each well and incubated for 4 h. The formazan crystals formed with MTT treatment were solubilized with DMSO and the optical density was measured at 570 nm with a reference wavelength of 630 nm using a microplate ELISA reader (SpectraMax^®^ i3x, Molecular Devices, Sunnyvale, CA). Percentage of inhibition was calculated as control-test/control*100 [19] and IC50 was estimated (S2 Methods).

### Cell cycle analysis

MCF7 cells were plated at a seeding density of 3×10^5^ cells/T25 flask and allowed to attach overnight. The cells were treated with three different concentrations (15, 20 and 25 mg/ml) of MEAD. Untreated cells served as negative control and doxorubicin (DOX) 50 μM was used as a positive control. After 24 h of incubation, the cells were trypsinized and washed with Dulbecco's phosphate buffered saline (PBS) containing no calcium and magnesium. The pellet was resuspended into single cell suspension and fixed with chilled 70% ethanol and kept overnight at -20°C temperature. Before analysis, the cells were centrifuged at 300 g for 5 min and washed twice with PBS. Cells were resuspended in 400 μl staining solution containing 0.5 μg/ml RNase A and 50 μg/ml propidium iodide (PI). Following 15 min of incubation in the dark, the cells were analyzed using the FACS Aria III flow cytometer (BD Biosc iences, San Jose, CA).

### Annexin V/PI assay

MCF7 cells were cultured overnight at a seeding density of 3×10^5^ cells/ T25 tissue culture flasks and treated with 15 mg/ml and 25 mg/ml MEAD for 48 h. Annexin V/PI assay was carried out according to the manufacturer's instructions. Briefly, the cells were harvested using 1% trypsin in PBS (Invitrogen, Carlsbad, CA), washed once with cold PBS and twice with 1X binding buffer. The final cell pellet was resuspended with 100 μl of freshly prepared PI (Sigma, St. Louis, MO) and annexin V-FITC solution (Trevigen Inc., Gaithersburg, MD) and incubated at room temperature in the dark for 15 min. Additionally 400 μl of binding buffer was added to the sample and analyzed within 30 min using flow cytometer (FACS Aria III, BD Biosciences, San Jose, CA). The percentage of early apoptotic, late apoptotic and necrotic cells was analyzed using BD FACS DIVA software.

### TUNEL (Terminal deoxynucleotidyl transferase dUTP nick end labeling) assay

APO-DIRECT^™^ Apoptosis Kit (Novus Biologicals, Littleton, CO) was used according to the manufacturer's instructions. MCF7 cells were cultured overnight at a seeding density of 3×10^5^ cells/T25 flask (Greiner Bio-One GmbH, Germany) with 15 mg/ml and 25 mg/ml concentrations of MEAD for 48 h. Cells were trypsinized, washed with DPBS, fixed with 1% paraformaldehyde (Sigma, St. Louis, MO) for 60 min at 4°C, washed twice with DPBS and then resuspended in ice-cold 70% ethanol and stored overnight at -20°C. They were then centrifuged and washed twice with wash buffer and incubated overnight with 50 μl of DNA labeling solution at RT in the dark. The reaction was stopped by washing twice with rinse buffer and the cells were incubated with PI/RNase solution in the dark at RT for 30 min before being analyzed using flow cytometer (FACS Aria III, BD Biosciences, San Jose, CA).

### Apoptotic protein expression analysis

MCF7 cells were seeded at a density of 3x10^5^ cells/T25 flask and treated with 15 mg/ml and 20 mg/ml MEAD and Bcl-2 inhibitor ABT-737 (10 μM) for 24 h. Treated cells were trypsinized, washed with cold DPBS and fixed with 4% formaldehyde for 10 min at 37°C. The cells were then washed twice with cold DPBS, permeabilized with 90% pre-chilled methanol for 30 min at RT, washed with incubation buffer (0.5% BSA) and incubated with primary rabbit polyclonal antibodies for Bax, p53 (Santa Cruz, Los Angeles, CA) and goat monoclonal antibody for Bcl-2 (Sigma, St. Louis, MO) at RT for 90 min. The cells were then washed thrice with incubation buffer and incubated with appropriate secondary antibodies [FITC conjugated bovine anti-rabbit polyclonal and mouse anti-goat Dylight 405] for 45 min at RT in the dark (S3 Methods). Finally, the cells were washed with incubation buffer, centrifuged and the pellet was resuspended in PBS before being analyzed using FACS Aria III flow cytometer (BD Biosciences, San Jose, CA).

### Caspase activation assay

Cleaved caspase 3 was detected using FITC conjugated active caspase 3 antibody (BD Biosciences, San Jose, CA) according to the manufacturer's instructions. MCF7 cells were seeded at a density of 3x10^5^ cells/T25 flask and treated with MEAD (15 mg/ml and 20 mg/ml) and Bcl-2 inhibitor ABT-737 (10 μM) for 24 h. The cells were trypsinized, washed twice with cold (1X) PBS and centrifuged at 300 g x 5 min. The cell pellet was resuspended in 0.5 ml BD Cytofix/Cytoperm solution and incubated for 20 min on ice. The cells were then washed twice with BD Perm/Wash buffer (1X) and incubated with 20 μl of FITC conjugated rabbit anti-caspase 3 antibody. Finally the cells were washed with wash buffer and resuspended in a 0.5 ml buffer and analyzed using FACS Aria III flow cytometer (BD Biosciences, San Jose, CA).

### Mitochondrial membrane potential analysis

Mitochondrial membrane potential (MMP) was analyzed in live cells using cationic lipophilic fluorochrome labelled dye, 5,5′,6,6′-Tetrachloro-1,1′,3,3′-tetraethylbenzimidazolylcarbo-cyanine chloride (JC-1) (BD Biosciences, San Jose, CA), as per manufacturer's instructions. JC-1 forms aggregates in the polarized healthy mitochondria (bearing membrane potential) where as in the apoptotic cells JC-1 forms monomers in cytoplasm. Briefly, MCF7 cells were treated with 15 mg/ml and 20 mg/ml MEAD for 24 h. ABT-737 (10 μM) used as control. The cells were then trypsinized, washed with (1X) DPBS and incubated with JC-1 solution for 20 min at 37°C. The cells were finally washed with assay buffer and analyzed using FACS Aria III flow cytometer (BD Biosciences, San Jose, CA).

### Quantitative Real Time-PCR (qRT-PCR)

MCF7 cells were seeded at a density of 2×10^5^cells/T25 tissue culture flasks and allowed to attach overnight. They were then treated with 15 mg/ml and 20 mg/ml of MEAD and cultured for 48 h. The untreated cells served as a control. Total RNA was extracted from the experimental and control cells using Pure Link^®^ RNA Mini Kit (Ambion^™^, ThermoFischer Scientific, Waltham, MA) which included DNase-I treatment protocol. RNA quantity and quality were measured using Nanodrop^™^ spectrophotometer (NanoDrop products, Wilmington, CA). First-strand cDNA synthesis was performed using random hexamers (High Capacity cDNA Reverse Transcription Kit, Applied Biosystems). Primer sequences (Table 1) used in the study were obtained from earlier published studies [20]. qRT-PCR analysis was performed using the ABI Step One Plus Real-Time PCR System (Applied Biosystems, Foster City, CA) using SYBR green dye master mix and relative quantification was performed using the comparative 2 –^ΔΔCt^ method.

**Table 1:** Real Time PCR Primers.

Genes	Primer type	Sequence
***Bax***	Forward	5’-TGCTTCAGGGTTTCATCCAG-3’
	Reverse	5’-GGCGGCAATCATCCTCTG-3’
***Bcl-2***	Forward	5’-GGCTGGGATGCCTTTGTG-3’
	Reverse	5’-CAGCCAGGAGAAATCAAACAGA-3’
***p53***	Forward	5’-CGGGATCCATGGAGGAGCCGCAGTCAGAT-3’
	Reverse	5’-CCGCTCGAGTTTCTGGGAAGGGACAGAAGA-3’
***β-Actin***	Forward	5’-GCACCACACCTTCTACAATG-3’
	Reverse	5’-TGCTTGCTGATCCACATCTG-3’
***Fas***	Forward	5’-TCTGGTTCTTACGTCTGTTGC-3’
	Reverse	5’-CTGTGCAGTCCCTAGCTTTCC-3’
***FasL***	Forward	5’-TGCCTTGGTAGGATTGGGC-3’
	Reverse	5’-GCTGGTAGACTCTCGGAGTTC-3’

### Statistical analysis

The differences observed between the control and treated groups for cell proliferation, cell cycle, apoptosis, caspase activation and MMP were analyzed using either One-way ANOVA or unpaired Student t-test (two-tailed) using GraphPad Prism 6 (GraphPad Software, San Diego, USA). The results were expressed as the Mean ± SEM (standard error of mean) from three different replicates and a value of p<0.05 was considered statistically significant.

## Results

### MEAD induced changes in cell morphology

**Fig 1:**
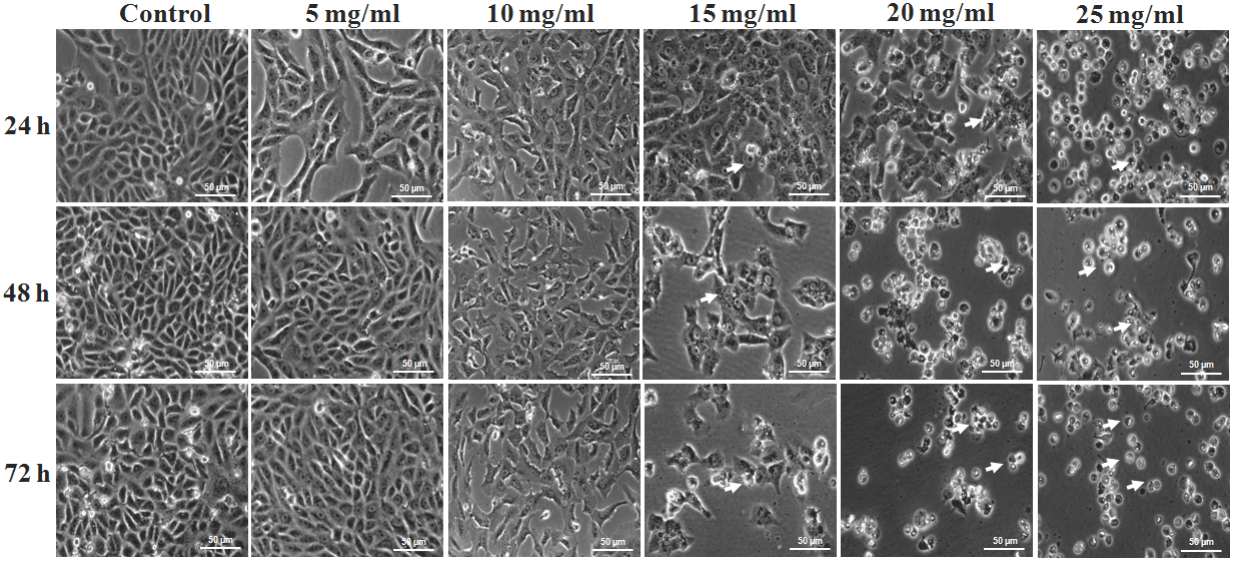
Phase contrast images of MCF7 cells. Phase contrast images of MCF7 cells showing morphological changes in a dose and time-dependent manner following treatment with MEAD at 5, 10, 15, 20 and 25 mg/ml. Prominent such as included cell shrinkage, membrane blebbing, cell fragmentations and detachment mimicking apoptosis were observed. There were also gross decrease in cell numbers with increase in time and concentration of MEAD. Arrows indicate dead cells which appears round and translucent (Magnification 100X).

Phase contrast microscopy of untreated MCF7 cells demonstrated their characteristic epithelial nature and prolific growth as a monolayer. The cells appeared ovoid with central large nucleus with one or two nucleoli. In contrast, the MEAD treated cells showed mild to moderate decrease in cell numbers (S1 Fig) that were both time and dose dependent resulting in the presence of very few live cells (Fig 1). Cell shrinkage was observed starting from 10 mg/ml of MEAD. Cytoplasmic condensation, membrane disruptions, detachment and cell death were most evident in MCF7 cells treated with 20 mg/ml and 25 mg/ml of MEAD (Fig 1).

### MEAD inhibited MCF7 proliferation

**Fig 2:**
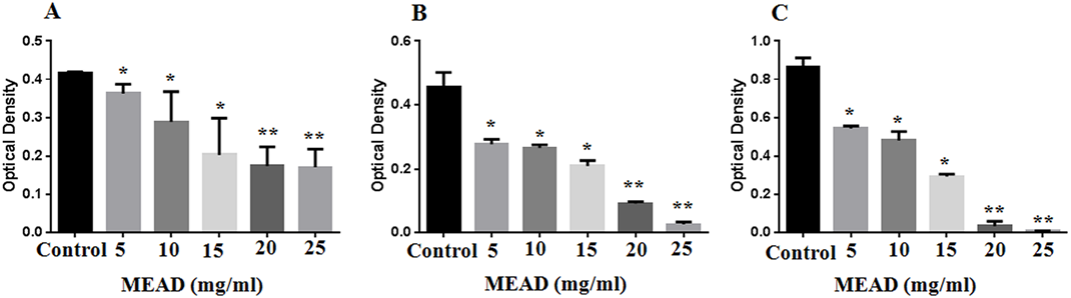
Inhibition of growth and proliferation of MCF7 cells. MTT assay of MCF7 cells treated with MEAD at different concentrations (5, 10, 15, 20 and 25 mg/ml) showed inhibition of cell proliferation compared to untreated controls in a dose dependent manner at 24 h, 48 h and 72 h respectively. (A) The percentage decrease in inhibition were (18.0 ± 40% to 60.80 ± 4.80%) at 24 h, (B) (33.70 ± 3.6% to 95.80 ± 2.0%) at 48 h and (C) (37.36 ± 1.60% to 96.0 ± 2.0%) at 72 h. These decreases in inhibitions were statistically significant. The values are expressed as mean ± SEM from triplicate samples of three independent experiments. * and ** indicate p<0.05 and p<0.001 respectively.

MTT assay demonstrated MCF7 growth inhibition following treatment with MEAD in a dose and time-dependent manner (Fig 2A–2C). Moderate decrease in cell numbers by 41.85 ± 4.6 percent was observed following incubation with 15 mg/ml MEAD for 48 h (Fig 2B) compared to the control. There was a decrease in cell numbers at 20 mg/ml concentration at 48 h and 72 h that ranged from 78.20 ± 1.34 to 96 ± 2.03 percentages respectively (Fig 2C). The cell proliferation showed a significant decrease (99.12 ± 0.08) in MCF7 cells treated with 25 mg/ml of MEAD at 72 h. The IC50 was determined to be 18.20 mg/ml (S2 and S3 Figs) for the 48 h duration. The decrease in proliferations were statistically significant (p<0.001).

### MEAD induced 'S' phase arrest in MCF7 cells

**Fig 3:**
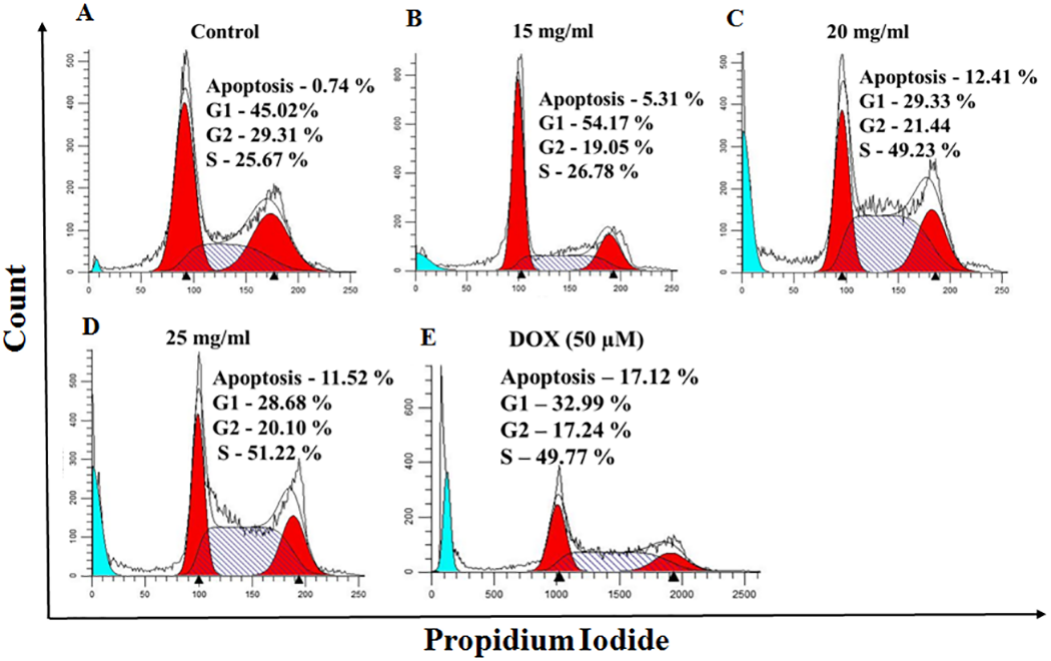
Cell cycle arrest in MCF7 cells. Representative cell cycle images of MCF7 cells after 24 h of treatment with MEAD at 15, 20 and 25 mg/ml MEAD. The histogram indicates 'S' phase arrest and decrease in cell numbers in 'G1' phase of cell cycle in a dose dependent manner. The sub-G1 phase which is indicative of apoptosis also showed increase in a dose dependent manner.

Cell cycle assay of MCF7 cells following treatment with different concentrations of MEAD (15, 20 and 25 mg/ml) showed altered cell cycle pattern compared to untreated controls (Fig 3A). The percentages of cells were 5.31%, 29.33%, 28.68% in 'G1' phase; 26.78%, 49.23%, 51.22% in 'S' phase and 19.05%, 21.44%, 20.10% in 'G2/M phase; for the concentrations 15, 20 and 25 mg/ml MEAD respectively (S4 Fig). There was a dose-dependent cell cycle arrest in the 'S' phase and a decrease in 'G1' phase of cell cycle in the treated group compared to the control (Fig 3B–3D).

### MEAD induced apoptosis in MCF7 cells

**Fig 4:**
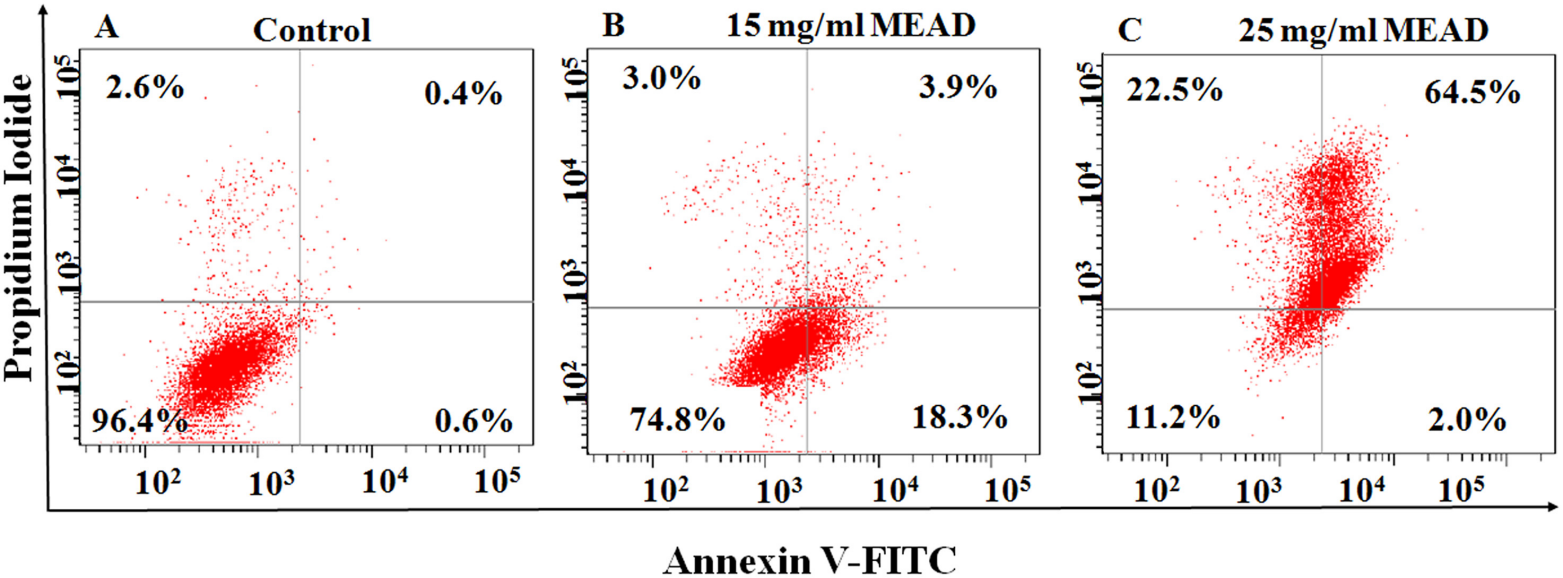
Apoptosis in MCF7 cells. Representative dot plot of the Annexin V-FITC and PI assay on MCF7 cells treated with 15 and 25 mg/ml of MEAD for 48 h showed increases in apoptotic cells compared to the control. These increases were statistically significant (p<0.05).

Annexin V-FITC and PI double staining of MCF7 cells treated with 15 mg/ml and 25 mg/ml concentrations of MEAD showed dose dependent increase in the number of apoptotic cells. The increases in total apoptotic cells were 21.45 ± 0.4% and 68.15 ± 1.55% for 15 mg/ml and 25 mg/ml of MEAD (S5 Fig) respectively compared to control 1% (Fig 4A–4C). These increase in apoptosis was statistically significant compared to the control (Fig 4D).

### MEAD increased TUNEL positive cells

**Fig 5:**
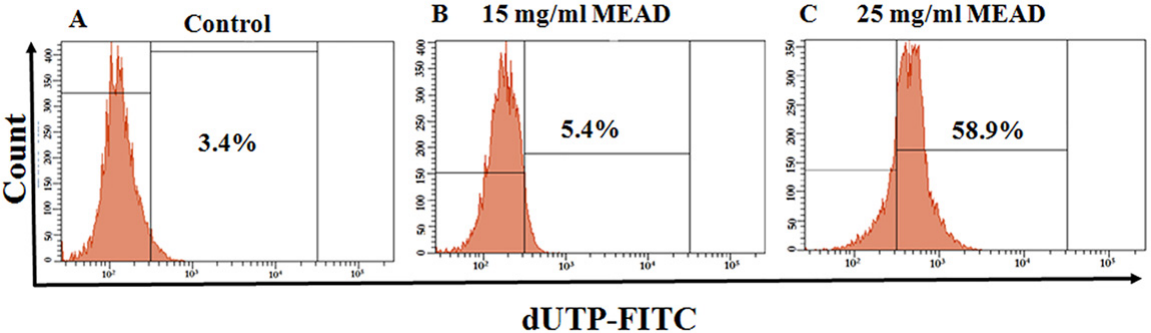
TUNEL Assay DNA fragmentation in MCF7 cells. MCF7 cells were treated for 48 h with 15 mg/ml and 25 mg/ml concentrations of MEAD respectively and incubated with DNA labeling solution containing dUTP-FITC and TdT polymerase overnight. FITC fluorescence represented the dUTP incorporation and DNA fragmentation. Representative dot plot image shows the fluorescence shift towards the left indicating the increase in TUNEL positive cells.

Flow cytometric evaluation of DNA fragmentation in MCF7 cells treated with 15 mg/ml and 25 mg/ml of MEAD for 48 h showed dose dependent increase in the numbers of TUNEL positive cells compared to the untreated control (Fig 5A). This increase in TUNEL positive cells were 3.0 ± 2.40% and 58.25 ± 0.65% for 15 mg/ml and 25 mg/ml of MEAD respectively (Fig 5B and 5C).

### MEAD increased expression of apoptotic proteins

**Fig 6:**
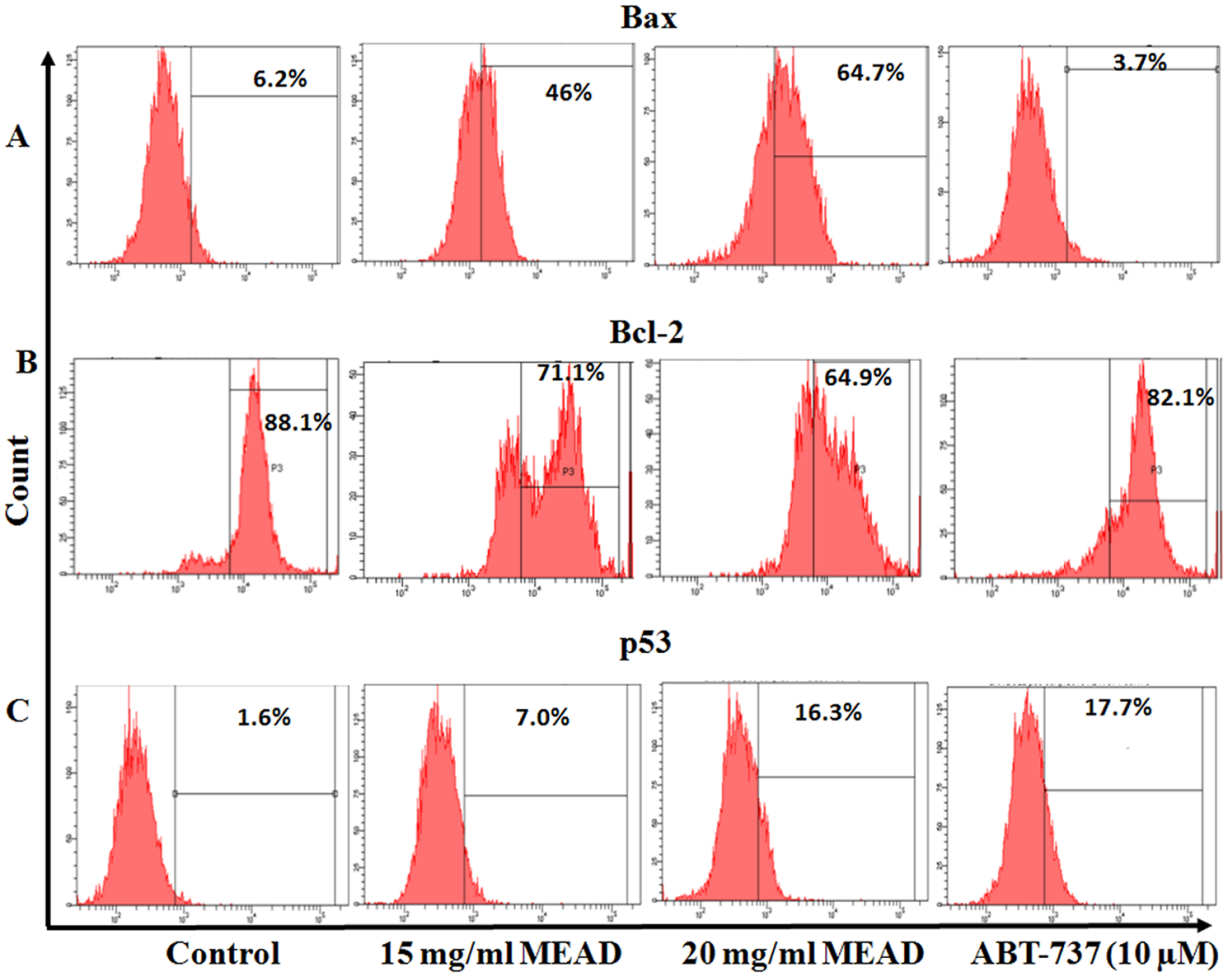
Apoptotic protein expression in MCF7 cells. MEAD up-regulate pro-apoptotic proteins (p53, Bax) and down-regulates the anti-apoptotic protein (Bcl-2) in MCF7 cells. MCF7 cells were treated with 15 and 20 mg/ml MEAD and Bcl-2 inhibitor ABT-737 (10 μM) concentrations for 24 h and staining is performed for p53, Bax and Bcl-2. The representative graphs are out of three independent flow cytometric analyses.

The expression levels of Bax, Bcl-2 and p53 were analyzed using flowcytometry (Fig 6). A dose dependent increase in the expression of p53 and Bax was observed in treated MCF7 cells as compared to the untreated control. There were more than 4 fold and 10 fold increase in the expression of p53 upon treatment of cells with 15 and 20 mg/ml MEAD respectively, as compared to the control. Bcl-2 inhibitor ABT-737, used as a positive control showed 11 fold increase in the expression of p53 as compared to the control (S6 Fig). Bax expression demonstrated more than 7 fold and 10 fold increase upon treatment of cells with 15 and 20 mg/ml MEAD respectively, as compared to the control. ABT-737 showed 1.6 fold decrease in Bax as compared to control. The expression of Bcl-2 decreased from 88.1% in untreated cells to 71.1% and 64% (Fig 6B) upon treatment with 15 and 20 mg/ml MEAD respectively. Treatment with ABT-737 showed 6% reduction in the expression of Bcl-2.

### MEAD increased Caspase 3 activity

**Fig 7:**
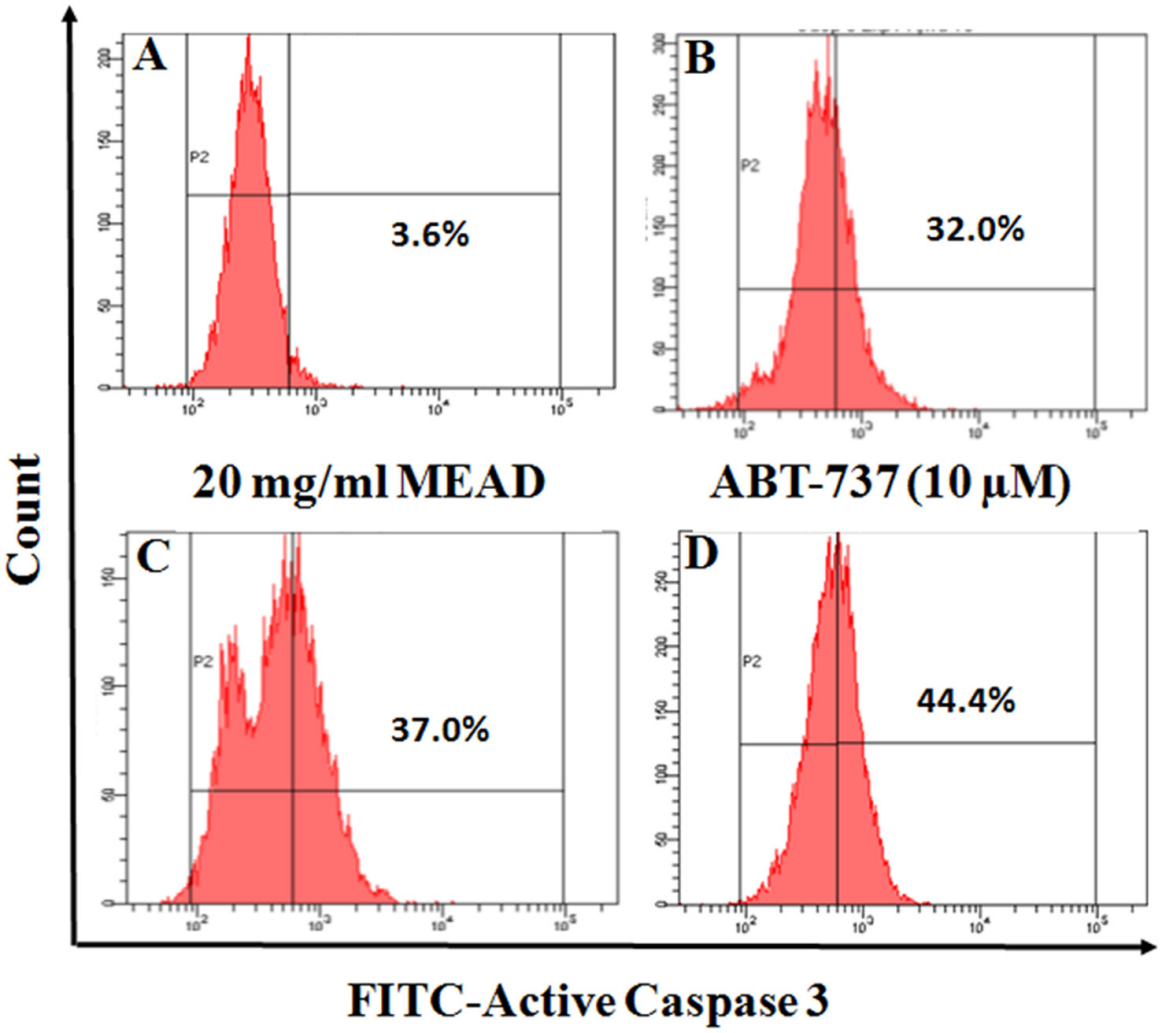
MEAD activates the Caspase 3 in MCF7 cells. MCF7 cells were treated with 15 and 20 mg/ml MEAD and Bcl-2 inhibitor ABT-737 (10 μM) for 24 h and stained with FITC-active caspase 3 antibody. The graphs represents one of the three independent flow cytometric analyses.

Treatment of MCF7 cells with MEAD showed a dose dependent increase in active Caspase 3 expression by FACS analysis. There was an increase in active caspase 3 staining (Fig 7B and 7C) by 32% and 37% following treatment with 15 mg/ml and 20 mg/ml MEAD respectively compared to the control (Fig 7A). Treatment with ABT-737 (10 μM) also demonstrated an increase in active caspase 3 expression.

### MEAD affects mitochondrial membrane potential in MCF7 cells

**Fig 8:**
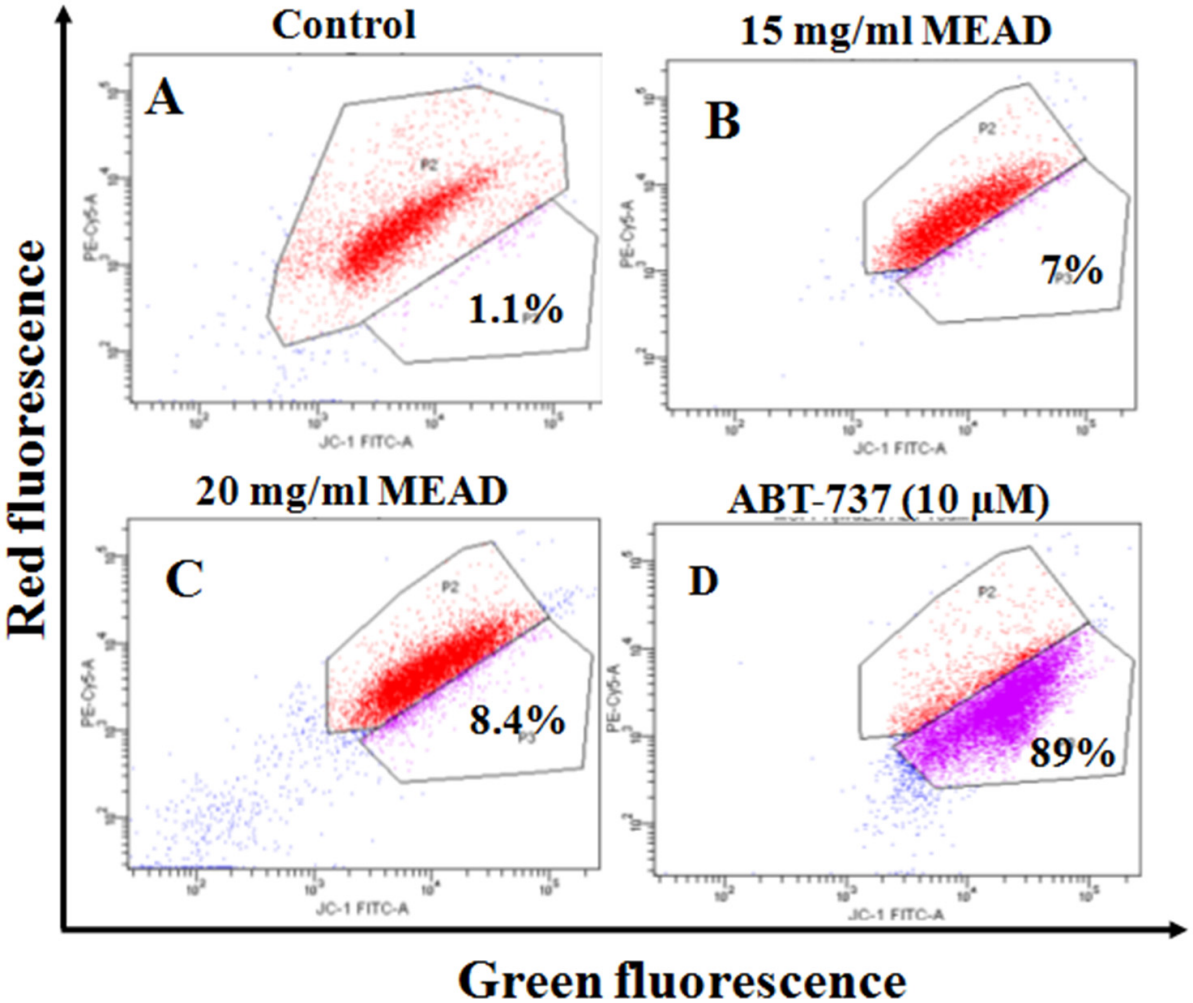
MEAD induces mild change in MMP in MCF7 cells. The scatter plot (representative of three independent experiments) for MCF7 cells showing JC-1 green fluorescence on the x-axis (indicator of loss of MMP) and PE-Cy5 red fluorescence on the y-axis (high MMP). MCF7 cells were treated with 15 and 20 mg/ml MEAD and Bcl-2 inhibitor ABT-737 (10 μM) for 24 h and stained with JC-1 and analyzed by flow cytometry.

Treatment of MCF7 cells with 15 and 20 mg/ml MEAD showed a mild increase of green fluorescent JC-1 8.4% and 7% (Fig 8B and 8C) respectively, compared to the control (Fig 8A). Treatment of cells with ABT-737 (10 μM), demonstrated 88% green fluorescent JC-1 staining, indicating a robust reduction of MMP (Fig 8D).

### MEAD up-regulated apoptotic gene expression

**Fig 9:**
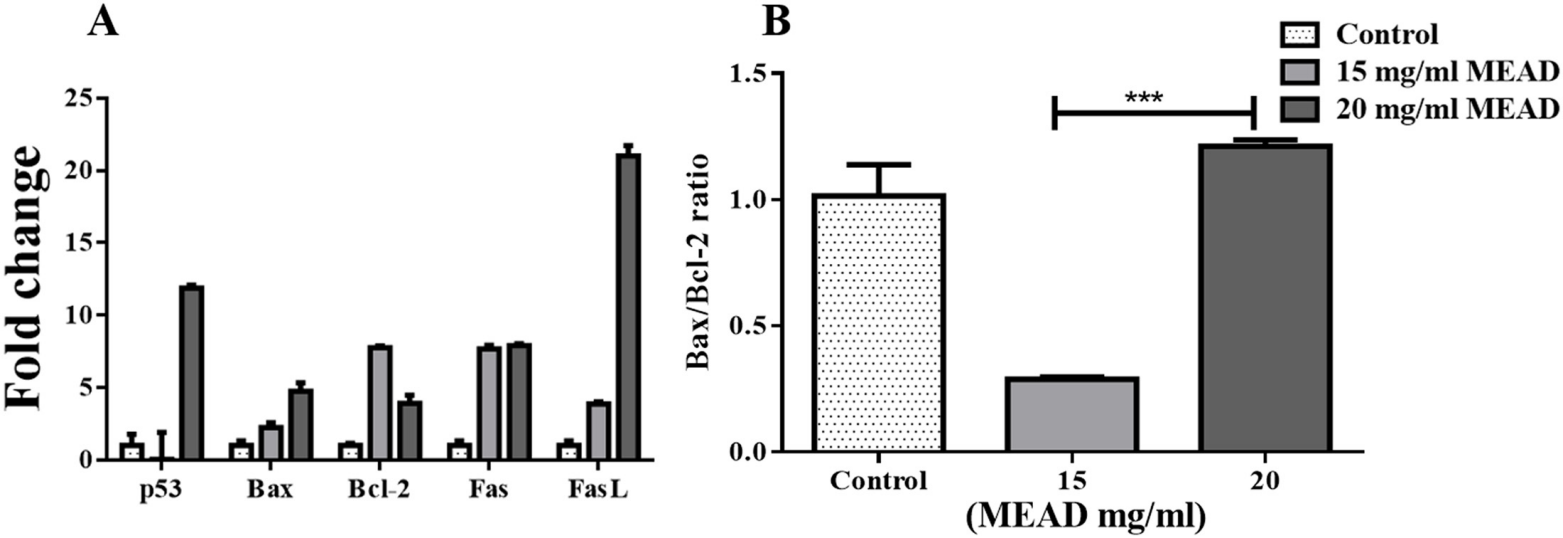
Apoptotic gene expression in MCF7 cells. (A) qRT-PCR analysis of MCF7 cells showing the *p53*, *Bax*, *Bcl-2*, *Fas* and *FasL* gene expression profile following treatment with 15 mg/ml and 20 mg/ml of MEAD for 48 h. *β-Actin* was used as an internal control and the data were calculated using the comparative 2 –ΔΔCt method. (B) The fold change ratio of *Bax*/*Bcl-2* gene expression got increased in a dose dependent manner. The values are expressed as mean ± SEM from triplicate samples of three independent experiments.

qRT-PCR analysis of the apoptotic related gene expression of MCF7 cells treated with 15 mg/ml and 25 mg/ml of MEAD for 48 h demonstrated an increase in *Bcl-2*, *p53*, *Fas* and *FasL* compared to the control. The fold increases were 18.07 ± 0.40, 42.87 ± 0.15 and 30.23 ± 0.49 for *p53*, *Fas* and *FasL* respectively and these increases were statistically significant (Fig 9A). The fold increases for *Bax* and *Bcl-2* were 6.40 ± 0.03 and 3.20 ± 0.25 respectively (Fig 9A). In addition, there was a dose-dependent increase in *Bax*/*Bcl-2* ratio from 0.29 ± 0.12 to 2.44 ± 0.12 (Fig 9B).

## Discussion

In this study, we have elucidated the anticancer effects of MEAD on MCF7 cells. MEAD inhibited the growth and proliferation of MCF7 cells. It also induced MCF7 cell death in a dose and time dependent manner. Natural products and their secondary metabolites have been demonstrated to induce apoptosis and/or modulate apoptotic pathways [20]. Many of the phytochemicals that exist in natural products/extracts such as flavonoids, aglycones, terepenoids and polyphenolic compounds are reported to have cancer inhibitory properties. Most importantly, in the last two decades some of them were subjected to clinical studies for various cancers including colon, liver, prostate, lung and breast cancer [21].

Cancer cells develop mechanism to regulate signaling pathways in order to escape host immune surveillance and avoid cell death, facilitating their prolonged survival [22]. The proliferative advantage conferred on these cells in turn promote their uncontrolled metastasis. Inhibition of their proliferation by induction of cell cycle arrest or apoptosis would be an advantage. Interestingly, in the present study, MEAD showed various morphological changes that were indicative of apoptosis. The cellular changes included membrane blebbing, cell contraction, cell fragmentation and loss of cell adherence leading on to cell death (Fig 1). In addition, both annexin V-FITC co-staining with PI as well as the TUNEL assays demonstrated annexin V-FITC and TUNEL positive cells following treatment with MEAD. These results are in support of our morphological findings and further point towards apoptosis of MCF7 cells by MEAD. Externalization of phosphatidylserine (PS) associated with loss of cell membrane asymmetry and fragmentation of nuclear DNA are considered to be the hallmarks of cells in later stages of apoptosis [23]. Our findings are similar to an earlier study where pancreatic cancer cells treated with myricetin showed an increase in annexin-V and TUNEL positive cells [24].

Mitochondria play a central role in both cell survival and cell death. Depolarization of mitochondrial membrane potential causes translocation of Bax from the cytosol to the outer mitochondrial membrane as well as the release of cytochrome c. These events then orchestrate apoptosome complex formation and activation of intrinsic apoptotic pathway [25–26]. In the present study, the MCF7 cells treated with MEAD, demonstrated mild MMP changes compared to the control (Fig 8) indicating that the apoptotic events are mediated through the intrinsic pathway. However, significant caspase 3 activation was also noted in MCF7 cells treated with MEAD indicating that additional pathways of apoptosis are involved. Activation of the caspases including caspase 8 and caspase 3 are implicated in ligand mediated extrinsic pathway of apoptosis [27]. Quercetin, apigenin luteolin and myricetin are some of the common constituents of Ajwa date extract [15]. Interestingly, these compounds are reported to cause cancer cell inhibition [28–30]. Independent studies have demonstrated the anticancer effects of quercetin, where human breast cancer cells (MDA-MB-231 and MCF7) were inhibited in a dose and time dependent manner [31]. Quercetin also caused cell cycle arrest, increased pro-apoptotic Bax protein, reduced MMP and activated caspase 3, 8 and 9 leading to apoptosis [31, 32].

Cell cycle regulation and cell death signaling pathways play a very important role in the developmental biology [32]. Dysregulation of cell signaling pathways leads to cancerous transformation and therefore serve as the potential targets for cancer therapeutics [32]. The cell cycle analysis in our study demonstrated 'S' phase arrest and decreased 'G1' phase following treatment with MEAD indicating that the extract and its components can bring about cell cycle arrest and inhibit the proliferation of cancer cells. MCF7 cell proliferation *in vitro* was inhibited in a dose and time dependent manner (Fig 2). This was in line with an earlier report where the methanolic extract of date pollen was found to have anti-proliferative effects on MCF7, Caco-2 and Hela-1 cell lines [33].

In line with our other results, the gene expression showed a significant increase in *p53*, *Bax*, *Fas* and *FasL* indicative of apoptotic cell death. p53 on stabilization and accumulation causes transcriptional regulation of different processes like cell cycle, apoptosis and DNA repair. *Bax*, *PUMA* and *NOXA* are up-regulated in apoptosis, while *Bcl-2* family genes are down-regulated [34]. p53 up-regulate the transcription of *Fas* and enhances its processing and release through the Golgi apparatus [35, 36]. Probably, the increase in p53 led to the transcriptional activation of other dependent pro-apoptotic genes such as *Fas*, *FasL*, and *Bax*. Increased expression of Bax and p53 protein in our study further support that apoptotic cell death of MCF7 could be due to p53 dependent mechanism. In addition, there was a decrease in anti-apoptotic *Bcl-2* and increase in the ratio of Bax/Bcl-2 transcription suggesting that MCF7 cell death following treatment with MEAD was activated by p53 mediated signaling leading to apoptosis. Quercetin, one of the metabolite of Ajwa date induces apoptosis by arresting cells in 'S' phase and 'G1' phase; expression of p53, P57 and Bax proteins; changes in mitochondrial membrane potential and activation of caspases in MCF7 cells [31]. This is in accordance with our results showing 'S' phase arrest and over expression of *p53* and increased *Bax/Bcl-2* ratio in MEAD treated MCF7 cells.

Plant extract have many different phytochemicals and metabolites, that may interact in a synergistic manner to exert their potential benefits to fight against the multifactorial and multistage carcinogenesis process [37]. Although it is important to purify the different components of the extract and identify their independent effects on both normal and cancer cell lines, at times the very process of purification may lead to the loss of these active compounds and hence their useful properties. Undue caution should therefore be exercised in isolation of individual components, as in some instances only the whole extract may actually be beneficial. This is emphasized by the fact that total extracts obtained from cranberry, pomegranate and Nigella sativa demonstrated much higher inhibitory effects in various cancers than their individual fractions [38–40]. Their beneficial effects were attributed to the synergistic effects of many individual components in the extract.

## Conclusion

MEAD induces cell cycle arrest and causes cell death *via* apoptosis by activating both intrinsic and extrinsic pathways in MCF7 cells. Unlike conventional chemotherapeutics, MEAD is observed to exert only mild to moderate beneficial effects against MCF7 cells. Purification of MEAD and the identification of individual components that are responsible for anti-cancer properties will pave way for the development of novel anticancer therapeutics. In addition, the aglycone metabolites of Ajwa dates such as Luteolin, myricetin, apigenin, quercetin and petunidin have been reported to induce apoptosis in cancers [41–44]. Given the folklore claims of cancer inhibitory properties of Ajwa date extract and our results on MCF7 cells, the date fruits could be added daily as a nutritional supplement for synergistic chemopreventive effects against breast cancer and other malignancies.

## Supporting Information

S1 Fig Phase contrast images of 3T3L1 cells. Phase contrast images of 3T3L1 cells following treatment with MEAD at 1, 5, 10, 15, 20, 25, 30, 50 and 100 mg/ml for 48h. There were decreases in the cell numbers with increasing concentrations of MEAD. (Magnification 100X).
(TIF)

S2 Fig IC50 calculation graph of 3T3L1 cells. 3T3L1 cells were treated with MEAD at 0, 1, 5, 10, 15, 20, 25, 30, 50 and 100 mg/ml concentrations for 48 h and MTT assay was performed. The data obtained was analyzed using log (inhibitor) vs. response—Variable slope (four parameters) analysis function with the help of Prism GraphPad 6.0 software. The IC50 value of 3T3L1 with MEAD is 50 mg/ml.
(TIF)

S3 Fig IC50 calculation graph of MCF7 cells. MCF7 cells were treated with MEAD at 0, 5, 10, 15, 20 and 25 mg/ml concentrations for 48 h and MTT assay was performed. The data obtained was analyzed using log (inhibitor) vs. response—Variable slope (four parameters) analysis function with the help of Prism GraphPad 6.0 software. The IC50 value of MCF7 with MEAD is 18.2 mg/ml.
(TIF)

S4 Fig Cell cycle arrest in MCF7 cells. MCF7 cells after 24 h of treatment with MEAD at 15, 20 and 25 mg/ml MEAD. The histogram indicates 'S' phase arrest and decrease in cell numbers in 'G1' phase of cell cycle in dose dependent manner.
(TIF)

S5 Fig Apoptosis in MCF7 cells. MCF cells treated with 15 and 25 mg/ml MEAD for 48 h. The histogram represents increase in Annexin V staining inactive of increase in apoptotic cells compared to the control. These increases were statistically significant (p>0.05).
(TIF)

S6 Fig P53 protein expression in MCF7 cells. MCF7 cell were treated with 15 mg/ml and 20 mg/ml MEAD for 24 h and immunocytochemistry was performed. Rabbit polyclonal primary antibody for P53 and FITC-labelled secondary antibody was used. Cells were additionally stained with DAPI nuclear stain. The upper row represent 15 mg/ml and lower row 20 mg/ml MEAD respectively.
(TIF)

S1 Methods 3T3L1 cells imaging. 3T3L1 cells were cultured in 24-well tissue culture plates (Beckton, Dickinson, Franklin Lanes, NJ) at a seeding density of 2×10^4^ cells/well. After overnight attachment, the cells were treated with MEAD at 0, 1, 5, 10, 15, 20, 25, 30, 50 and 100 mg/ml concentrations for 48 h. The cells were cultured under standard culture conditions of 37°C in a humidified atmosphere of 95% air and 5% CO_2_. Changes in cell morphology were imaged using inverted phase contrast microscope (Nikon Instruments, Tokyo Japan).
(DOCX)

S2 Methods IC50 Calculation of MEAD on 3T3L1 and MCF7 cell lines. 3T3L1 and MCF7 cells were cultured in 96-well tissue culture plates (Beckton, Dickinson, Franklin Lanes, NJ) at a seeding density of 5×10^3^ cells/well. After overnight attachment, the MCF7 cells were treated with MEAD at 0, 5, 10, 15, 20 and 25 mg/ml concentrations and the 3T3L1 cells were treated with MEAD at 0, 1, 5, 10, 15, 20, 25, 30, 50 and 100 mg/ml concentrations for 48 h and MTT assay was done. The optical densities were analyzed using log (inhibitor) vs. response—Variable slope (four parameters) analysis function with the help of Prism GraphPad 6.0 software to calculate the half maximal inhibitory concentration (IC50) value.
(DOCX)

S3 Methods Immunocytochemistry. MCF7 cells were plated at a seeding density of 2×10^4^cells/well in a 24-well plate and allowed to attach overnight. Fresh medium containing 20 mg/ml and 25 mg/ml MEAD was added and the cells were incubated for 48 h. Following incubation the cells were washed twice with cold PBS and fixed with 4% formaldehyde for 20 min. The cells were then washed and permeabilized with 0.25% triton X-100 for 30 min at RT. The cells were washed twice with cold PBS and blocked with 1% BSA (bovine serum albumin), followed by overnight incubation with primary antibody P53 (Santa Cruz, Los Angeles, CA) at 1:200 dilution at 4°C. The cells were then washed and incubated with FITC labelled anti-rabbit polyclonal secondary antibody (1:500) (Santa Cruz, Los Angeles, CA) in dark for 1 h at RT. The cells were stained with 1 μg/ml DAPI for 1 min and observed under ZEISS LSM 780 Laser Scanning Microscope (Carl Zeiss, Oberkochen).
(DOCX)
