# Supplementary figures and images for: Ajwa Date (Phoenix dactylifera L.) Extract Inhibits Human Breast Adenocarcinoma (MCF7) Cells In Vitro by Inducing Apoptosis and Cell Cycle Arrest
Source: PLoS One. 2016 Jul 21;11(7):e0158963. doi: 10.1371/journal.pone.0158963 (PMC4956039; doi:10.1371/journal.pone.0158963)

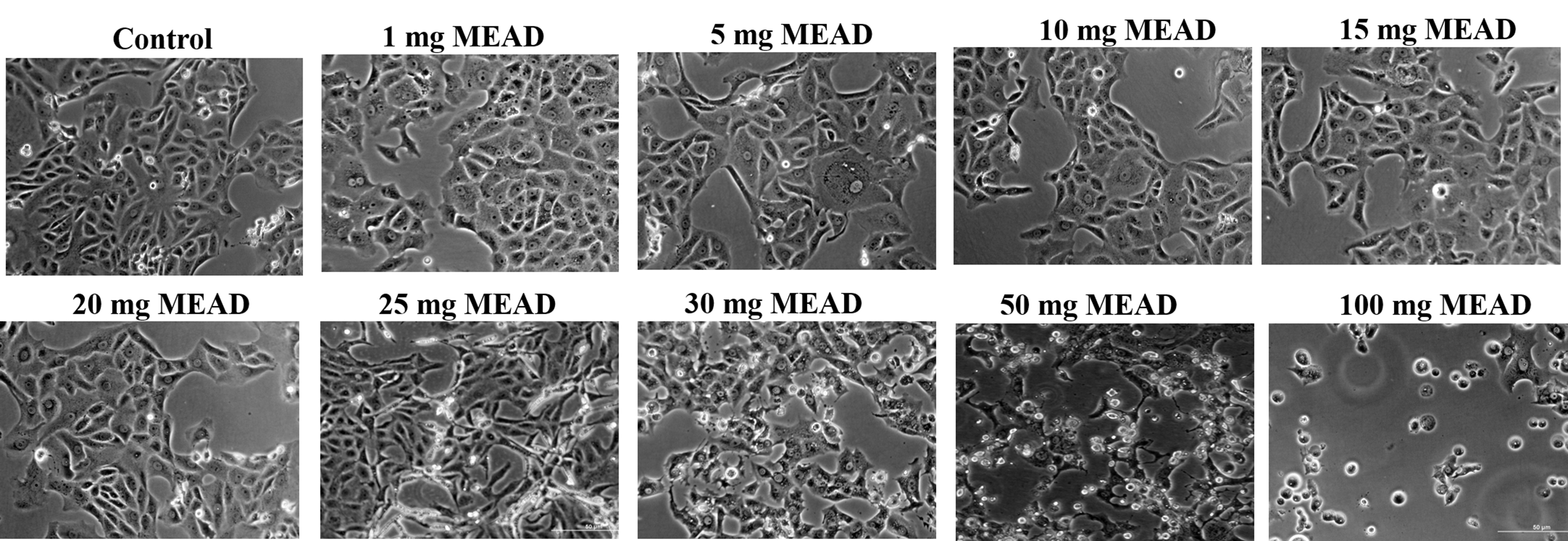

Supplement: S1 Fig — Phase contrast images of 3T3L1 cells following treatment with MEAD at 1, 5, 10, 15, 20, 25, 30, 50 and 100 mg/ml for 48h. There were decreases in the cell numbers with increasing concentrations of MEAD. (Magnification 100X). (TIF) [file pone.0158963.s001.tif]

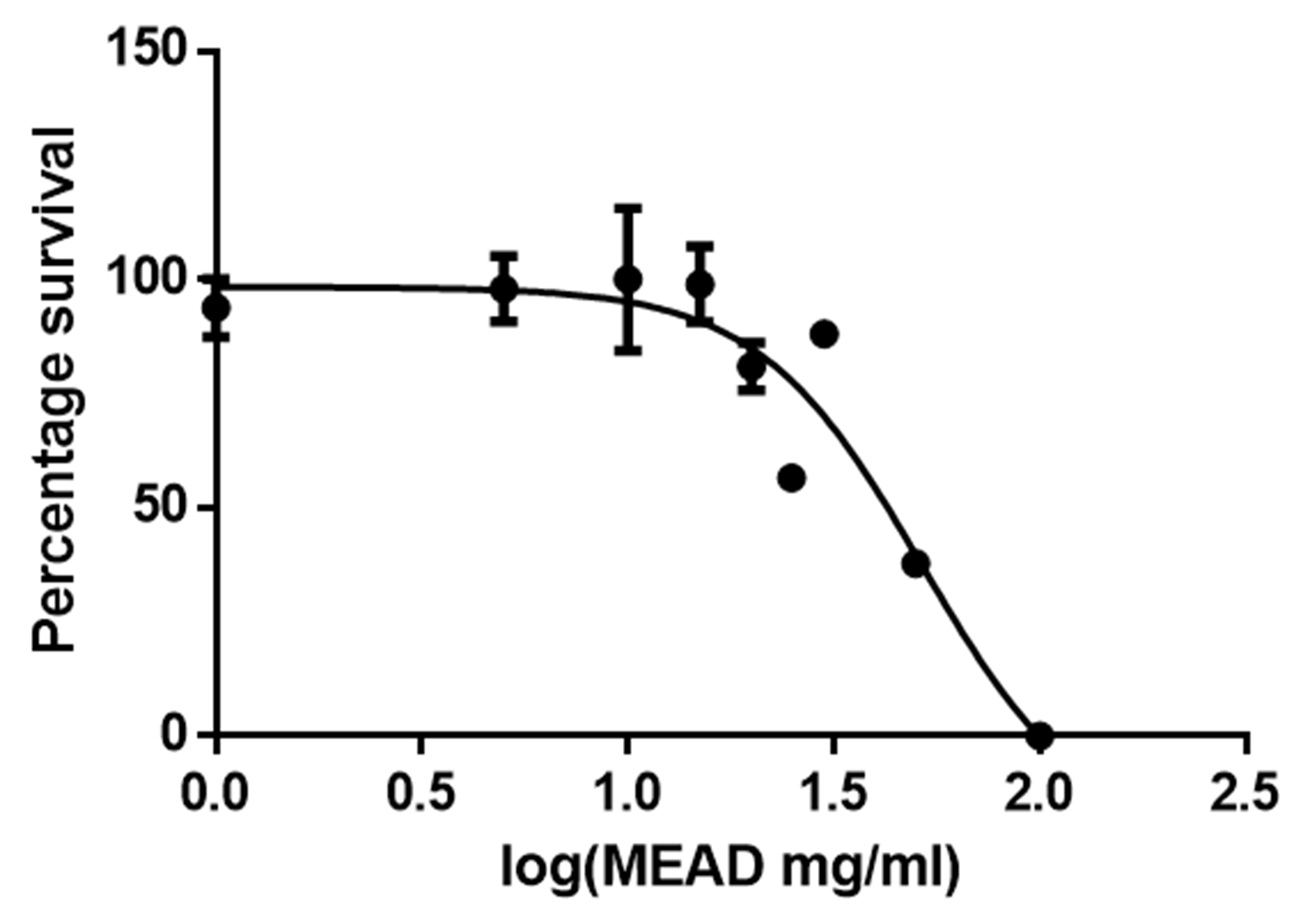

Supplement: S2 Fig — 3T3L1 cells were treated with MEAD at 0, 1, 5, 10, 15, 20, 25, 30, 50 and 100 mg/ml concentrations for 48 h and MTT assay was performed. The data obtained was analyzed using log (inhibitor) vs. response—Variable slope (four parameters) analysis function with the help of Prism GraphPad 6.0 software. The IC50 value of 3T3L1 with MEAD is 50 mg/ml. (TIF) [file pone.0158963.s002.tif]

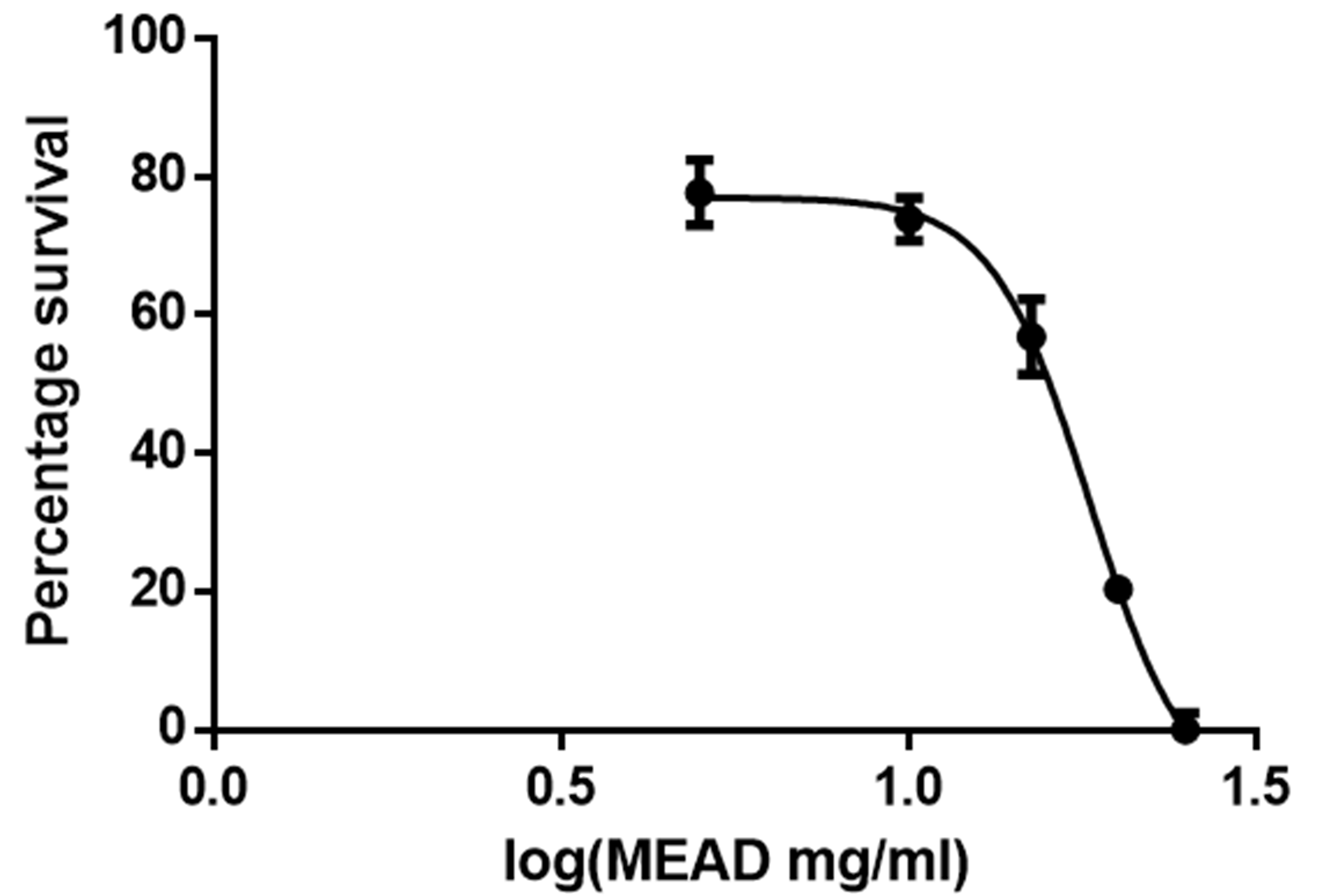

Supplement: S3 Fig — MCF7 cells were treated with MEAD at 0, 5, 10, 15, 20 and 25 mg/ml concentrations for 48 h and MTT assay was performed. The data obtained was analyzed using log (inhibitor) vs. response—Variable slope (four parameters) analysis function with the help of Prism GraphPad 6.0 software. The IC50 value of MCF7 with MEAD is 18.2 mg/ml. (TIF) [file pone.0158963.s003.tif]

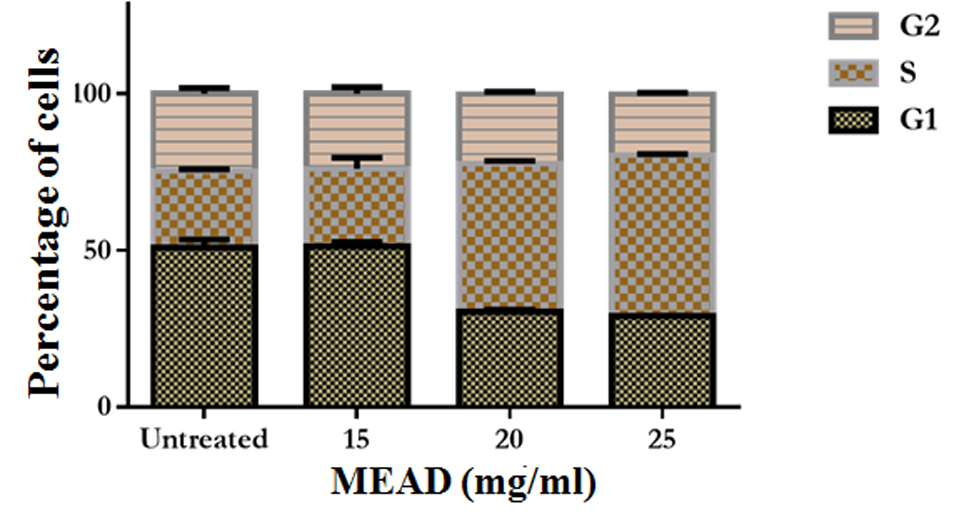

Supplement: S4 Fig — MCF7 cells after 24 h of treatment with MEAD at 15, 20 and 25 mg/ml MEAD. The histogram indicates 'S' phase arrest and decrease in cell numbers in 'G1' phase of cell cycle in dose dependent manner. (TIF) [file pone.0158963.s004.tif]

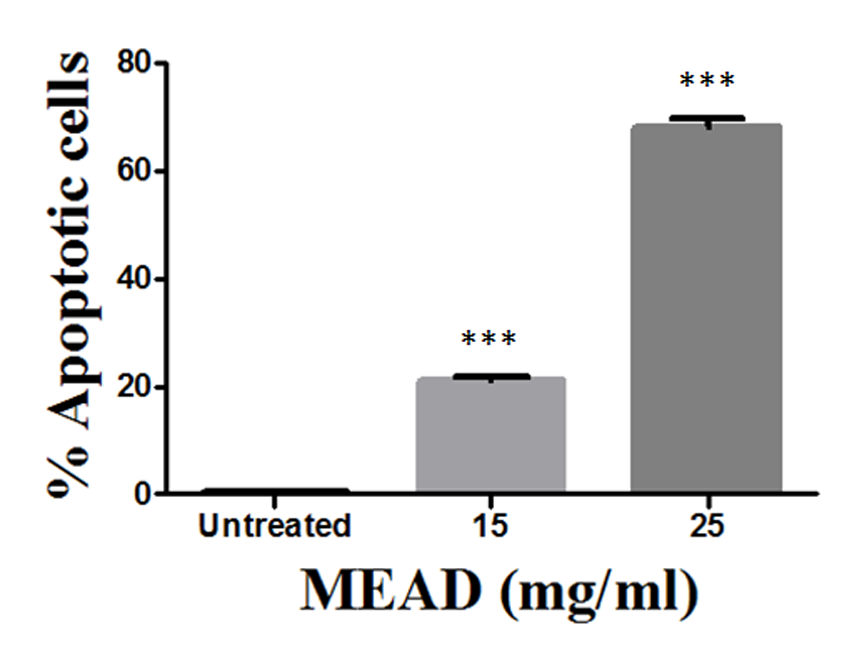

Supplement: S5 Fig — MCF cells treated with 15 and 25 mg/ml MEAD for 48 h. The histogram represents increase in Annexin V staining inactive of increase in apoptotic cells compared to the control. These increases were statistically significant (p>0.05). (TIF) [file pone.0158963.s005.tif]

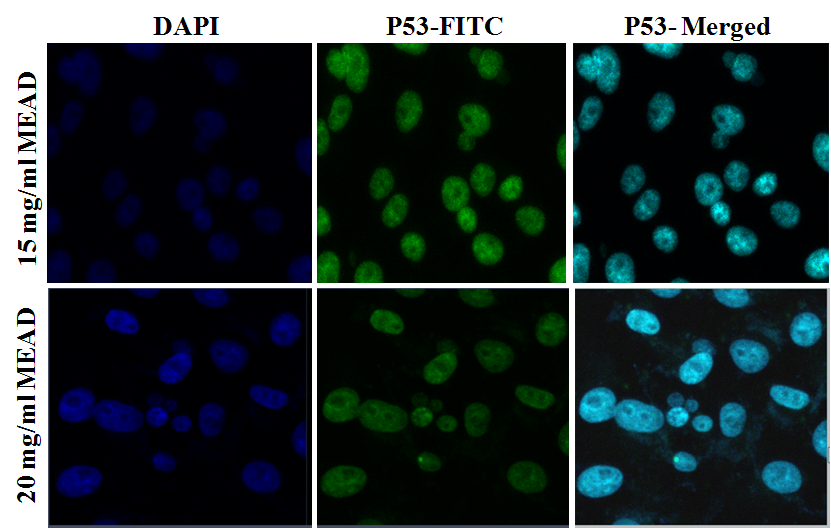

Supplement: S6 Fig — MCF7 cell were treated with 15 mg/ml and 20 mg/ml MEAD for 24 h and immunocytochemistry was performed. Rabbit polyclonal primary antibody for P53 and FITC-labelled secondary antibody was used. Cells were additionally stained with DAPI nuclear stain. The upper row represent 15 mg/ml and lower row 20 mg/ml MEAD respectively. (TIF) [file pone.0158963.s006.tif]
