# Supplementary material for: Ajwa Date (Phoenix dactylifera L.) Extract Inhibits Human Breast Adenocarcinoma (MCF7) Cells In Vitro by Inducing Apoptosis and Cell Cycle Arrest
Source: PLoS One. 2016 Jul 21;11(7):e0158963. doi: 10.1371/journal.pone.0158963 (PMC4956039; doi:10.1371/journal.pone.0158963)
Supplement: S1 Methods — 3T3L1 cells were cultured in 24-well tissue culture plates (Beckton, Dickinson, Franklin Lanes, NJ) at a seeding density of 2×10^4^ cells/well. After overnight attachment, the cells were treated with MEAD at 0, 1, 5, 10, 15, 20, 25, 30, 50 and 100 mg/ml concentrations for 48 h. The cells were cultured under standard culture conditions of 37°C in a humidified atmosphere of 95% air and 5% CO2. Changes in cell morphology were imaged using inverted phase contrast microscope (Nikon Instruments, Tokyo Japan). (DOCX) [file pone.0158963.s007.docx]

**S1 Methods. 3T3L1 cells imaging.** 3T3L1 cells were cultured in 24-well tissue culture plates (Beckton, Dickinson, Franklin Lanes, NJ) at a seeding density of 2×10^4^ cells/well. After overnight attachment, the cells were treated with MEAD at 0, 1, 5, 10, 15, 20, 25, 30, 50 and 100 mg/ml concentrations for 48 h. The cells were cultured under standard culture conditions of 37°C in a humidified atmosphere of 95% air and 5% CO_2_. Changes in cell morphology were imaged using inverted phase contrast microscope (Nikon Instruments, Tokyo Japan).
