# Supplementary material for: Ajwa Date (Phoenix dactylifera L.) Extract Inhibits Human Breast Adenocarcinoma (MCF7) Cells In Vitro by Inducing Apoptosis and Cell Cycle Arrest
Source: PLoS One. 2016 Jul 21;11(7):e0158963. doi: 10.1371/journal.pone.0158963 (PMC4956039; doi:10.1371/journal.pone.0158963)
Supplement: S2 Methods — 3T3L1 and MCF7 cells were cultured in 96-well tissue culture plates (Beckton, Dickinson, Franklin Lanes, NJ) at a seeding density of 5×10^3^ cells/well. After overnight attachment, the MCF7 cells were treated with MEAD at 0, 5, 10, 15, 20 and 25 mg/ml concentrations and the 3T3L1 cells were treated with MEAD at 0, 1, 5, 10, 15, 20, 25, 30, 50 and 100 mg/ml concentrations for 48 h and MTT assay was done. The optical densities were analyzed using log (inhibitor) vs. response—Variable slope (four parameters) analysis function with the help of Prism GraphPad 6.0 software to calculate the half maximal inhibitory concentration (IC50) value. (DOCX) [file pone.0158963.s008.docx]

**S2 Methods. IC50 Calculation of MEAD on 3T3L1 and MCF7 cell lines.** 3T3L1 and MCF7 cells were cultured in 96-well tissue culture plates (Beckton, Dickinson, Franklin Lanes, NJ) at a seeding density of 5×10^3^ cells/well. After overnight attachment, the MCF7 cells were treated with MEAD at 0, 5, 10, 15, 20 and 25 mg/ml concentrations and the 3T3L1 cells were treated with MEAD at 0, 1, 5, 10, 15, 20, 25, 30, 50 and 100 mg/ml concentrations for 48 h and MTT assay was done. The optical densities were analyzed using log (inhibitor) vs. response - Variable slope (four parameters) analysis function with the help of Prism GraphPad 6.0 software to calculate the half maximal inhibitory concentration (IC50) value.
