# Supplementary material for: Ajwa Date (Phoenix dactylifera L.) Extract Inhibits Human Breast Adenocarcinoma (MCF7) Cells In Vitro by Inducing Apoptosis and Cell Cycle Arrest
Source: PLoS One. 2016 Jul 21;11(7):e0158963. doi: 10.1371/journal.pone.0158963 (PMC4956039; doi:10.1371/journal.pone.0158963)
Supplement: S3 Methods — MCF7 cells were plated at a seeding density of 2×10^4^cells/well in a 24-well plate and allowed to attach overnight. Fresh medium containing 20 mg/ml and 25 mg/ml MEAD was added and the cells were incubated for 48 h. Following incubation the cells were washed twice with cold PBS and fixed with 4% formaldehyde for 20 min. The cells were then washed and permeabilized with 0.25% triton X-100 for 30 min at RT. The cells were washed twice with cold PBS and blocked with 1% BSA (bovine serum albumin), followed by overnight incubation with primary antibody P53 (Santa Cruz, Los Angeles, CA) at 1:200 dilution at 4°C. The cells were then washed and incubated with FITC labelled anti-rabbit polyclonal secondary antibody (1:500) (Santa Cruz, Los Angeles, CA) in dark for 1 h at RT. The cells were stained with 1 μg/ml DAPI for 1 min and observed under ZEISS LSM 780 Laser Scanning Microscope (Carl Zeiss, Oberkochen). (DOCX) [file pone.0158963.s009.docx]

**S3 Methods: Immunocytochemistry.** MCF7 cells were plated at a seeding density of 2×10^4^cells/well in a 24-well plate and allowed to attach overnight. Fresh medium containing 20 mg/ml and 25 mg/ml MEAD was added and the cells were incubated for 48 h. Following incubation the cells were washed twice with cold PBS and fixed with 4% formaldehyde for 20 min. The cells were then washed and permeabilized with 0.25% triton X-100 for 30 min at RT. The cells were washed twice with cold PBS and blocked with 1% BSA (bovine serum albumin), followed by overnight incubation with primary antibody P53 (Santa Cruz, Los Angeles, CA) at 1:200 dilution at 4°C. The cells were then washed and incubated with FITC labelled anti-rabbit polyclonal secondary antibody (1:500) (Santa Cruz, Los Angeles, CA) in dark for 1 h at RT. The cells were stained with 1 µg/ml DAPI for 1 min and observed under ZEISS LSM 780 Laser Scanning Microscope (Carl Zeiss, Oberkochen).
